# All-endoscopic autologous suspension fixation of semitendinosus tendon and gracilis tendon for insertional chronic Achilles tendon rupture: operative technique and outcomes

**DOI:** 10.1007/s00264-026-06805-3

**Published:** 2026-04-14

**Authors:** Hao Han, Zexiang Lv, Buqing Chang, Jie Li, Aiguo Wang, Yunjia Hao

**Affiliations:** Xuzhou Central Hospital, Xuzhou Clinical School of Nanjing Medical University, Xuzhou, China

**Keywords:** Chronic Achilles tendon rupture, Autologous tendon suspension fixation, Endoscopically assisted technique, Surgical treatment, ATRS

## Abstract

**Purpose:**

Acute Achilles tendon rupture with delayed treatment more than four weeks is referred to as chronic, which can lead to severe functional impairment. The literature advocates surgical reconstruction to restore ankle joint push-off strength. This study aims to introduce the technique and clinical outcomes of endoscopic autologous tendon suspension fixation for chronic insertional Achilles tendon rupture.

**Methods:**

Twenty-two patients (16 males, 6 females) with a mean age (range) of 49.2 ± 10.3 (30–67) years underwent Achilles tendon reconstruction surgery using all-endoscopic autologous tendon suspension fixation. Patients were evaluated at the last follow-up, with assessment indicators including Visual analogue scale (VAS), American Orthopaedic Foot & Ankle Society Ankle Hindfoot Scale (AOFAS-AH), Achilles tendon total rupture score (ATRS), Foot and Ankle Ability Measure (FAAM), Range of motion (ROM) and maximum calf circumference.

**Results:**

All patients successfully completed the surgery, with an operation time of 62.91 ± 8.82 (45–80) min, intraoperative blood loss of 15 (5–35) mL, and all surgical approaches healed in one stage, with no damage to important structures such as blood vessels, nerves, and tendons during the operation. Twenty-two patients were followed up for 16.23 ± 2.94 (12–23) months. Two patients reported weakness in single-leg heel raises, which subsequently improved with heel raise exercises. At the last follow-up, the AOFAS-AH score improved from 60.64 ± 8.83 (45–77) preoperatively to 94.18 ± 3.91 (88–100), while the ATRS score increased from 45.59 ± 5.85 (35–57) preoperatively to 93.18 ± 4.68 (83–100), and the VAS score decreased from 6 (1) to 1 (0), with all differences being statistically significant. Similarly, the FAAM- Activity of Daily Living (FAAM-ADL) score increased from 44.73 ± 8.79 (30–59) to 90.95 ± 4.62 (83–99), and the FAAM- -Sports (FAAM-S) score increased from 43.55 ± 7.14 (31–55) to 88.27 ± 8.18 (74–99). All differences were statistically significant. (all *P* < 0.001). The dorsiflexion angle of the affected side ankle joint (13.2 ± 1.9°), plantar flexion angle of the ankle joint (44.3 ± 1.6°), and maximum calf circumference (35.6 ± 1.5 cm) were compared with the healthy side (13.3 ± 1.9°, 44.5 ± 1.7°, 35.6 ± 1.6 cm), and there was no statistically significant difference (all *P* > 0.05). According to the Arner-Lindholm scoring assessment: excellent in 19 cases, good in three cases, with an excellent and good rate of 100% (22/22).

**Conclusion:**

This study demonstrates that all-endoscopic autologous suspension fixation achieves satisfactory outcomes in patients with chronic Achilles tendon ruptures. This technique effectively restores distal ruptures, making it a viable option for Achilles tendon reconstruction.

## Introduction

The Achilles tendon is the strongest muscle tendon in the human body, transmitting the contraction force of the gastrocnemius muscle to complete complex movements such as gait, jumping, and running [1, 2]. Chronic Achilles tendon ruptures are commonly caused by misdiagnosis, neglect, or failure to recognize acute rupture. Acute Achilles tendon rupture is a common missed and misdiagnosed condition, and patients may experience chronic rupture four to six weeks after the injury [3, 4]. The tendon ends frequently retracted, accompanied by significant dysfunction, and surgical treatment should be surgically undertaken unless there are severe surgical contraindications [5, 6]. Recent studies have reported the use of autologous semitendinosus and gracilis muscle tendons to treat chronic Achilles tendon rupture, demonstrating favourable clinical efficacy [7]. This method does not damage the primary tendon and results in high Achilles tendon strength postoperatively. However, as a traditional open surgical procedure, it involves a large incision and is associated with a high incidence of postoperative wound complications [8]. Endoscopic technology offers the characteristics of minimal trauma, rapid recovery, and precise repair, and is gaining increasing popularity [9, 10]. The purpose of this study is to describe the clinical and radiological outcomes of endoscopic autologous tendon suspension fixation in patients with chronic Achilles tendon rupture.

## Method and materials

### Patient demographic information

The present study was performed according to the STROBE Statement [11]. It was conducted as a retrospective, consecutive case series with a single follow-up investigation.

**Fig. 1:**
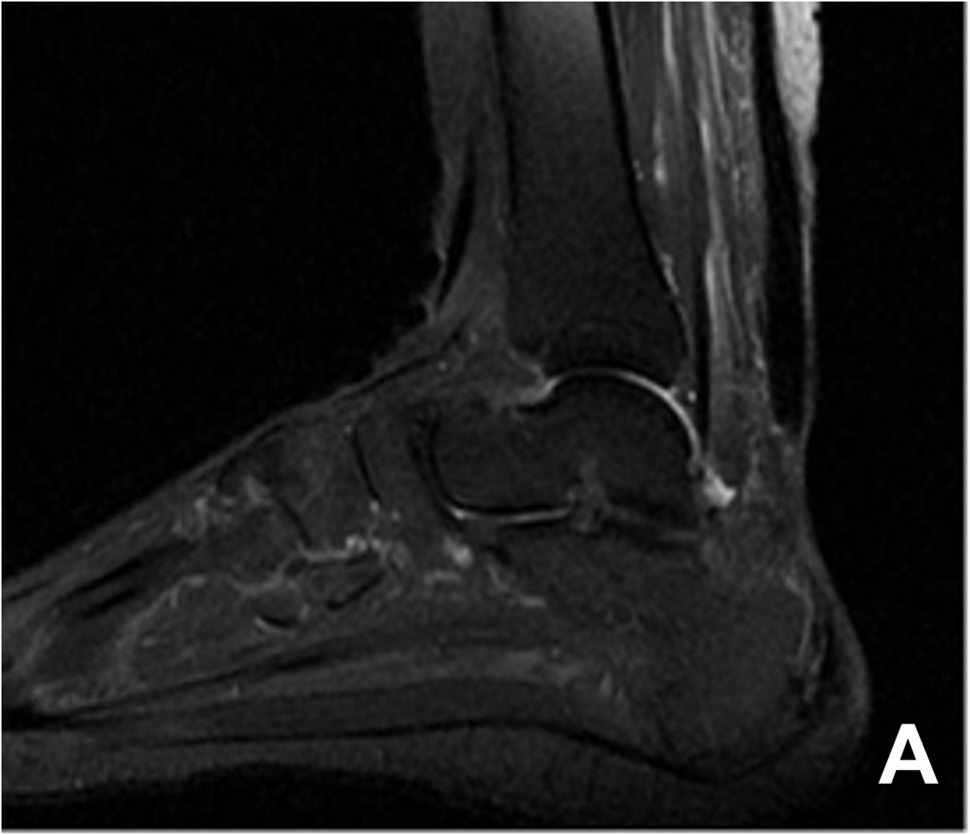
**A** 52-year-old female patient, presented with preoperative MRI images showing a complete rupture of the Achilles tendon at the insertion point

This study retrospectively analyzed the clinical data of patients with chronic insertional Achilles tendon rupture who underwent all-endoscopic surgery between January 2021and February 2024 and had a postoperative follow-up period exceeding 12 months. All patients underwent MRI examination before surgery to clarify the site of Achilles tendon rupture, the extent of the Achilles tendon defect, and X-ray examination to measure the Fowler-Phillip angle, clarifying the condition of the calcaneal posterior superior prominence and the bone quality at the Achilles tendon insertion site (Fig. 1). Inclusion criteria: (1) Chronic insertional Achilles tendon rupture confirmed by MRI, defined as injury duration > 4 weeks; (2) Rupture site located within 2 cm proximal to the calcaneal tuberosity; (3) Tendon defect > 5 cm (Myerson Type III) after thorough debridement; (4) Unilateral closed injury; (5) Complete preoperative and postoperative data with follow-up ≥ 12 months. Exclusion criteria: (1) Intraoperative minimally invasive surgery converted to open surgery; (2) Patients with poor local soft tissue conditions who cannot undergo endoscopic procedures; (3) Incomplete data or follow-up time < 12 months.

Twenty-two patients were included in this study, all presenting with unilateral rupture, with 17 cases on the right side and five cases on the left; 16 males and six females. The average age was (49.2 ± 10.3) years (range: 30–67 years); the average time from injury to surgery was (14.7 ± 5.6) weeks (range: 6–28 weeks). Causes of injury: seven cases of violent injury during sports moments, ten cases of sprains during walking, and five cases with no obvious violent factors.

This study has been approved by the Ethics Committee of Xuzhou Central Hospital (Grant No: XZXY-LJ-20190111-027) and complies with the requirements of the Declaration of Helsinki. All patients have signed informed consent forms. The patient demographics are presented in Table 1. All surgeries were performed by a surgeon (Yunjia Hao) with extensive experience in endoscopic surgery.

**Table 1:** Demographic characteristics of the patients included

Patient demographics (*n* = 22)	Mean ± SD median (range)
Age(year)	49.23 ± 10.32
Gender	
Male	16(72.7%)
Female	6(27.3%)
Location	
Left	5(22.7%)
Right	17(77.3%)
Cause of injury	
Sports-related acute injuries	7(31.8%)
Sprain during walking	10(45.5%)
No obvious violent elements	5(22.7%)
Time from injury to surgery(weeks)	14.68 ± 5.59
Operative time(mins)	62.91 ± 8.82
Intraoperative blood loss(ml)	15(11)
Length of Achilles tendon defect(cm)	6.57 ± 0.89
Autologous tendon length(cm)	23.55 ± 2.13
Length of the folded four-strand tendon(cm)	7(3)
Follow-time(months)	16.23 ± 2.94

### Surgical technique

The patient was placed in the prone position with a thigh tourniquet under spinal anesthesia or general anaesthesia. The hindfoot endoscopic portals (posteromedial and posterolateral) were located at the same level of the fibular tip or slightly distal to both sides of the Achilles tendon. The posterolateral portal was served as the working portal, while the posteromedial portal was equipped with a 4.0 mm 30° arthroscope as the visualization portal.

**Fig. 2:**
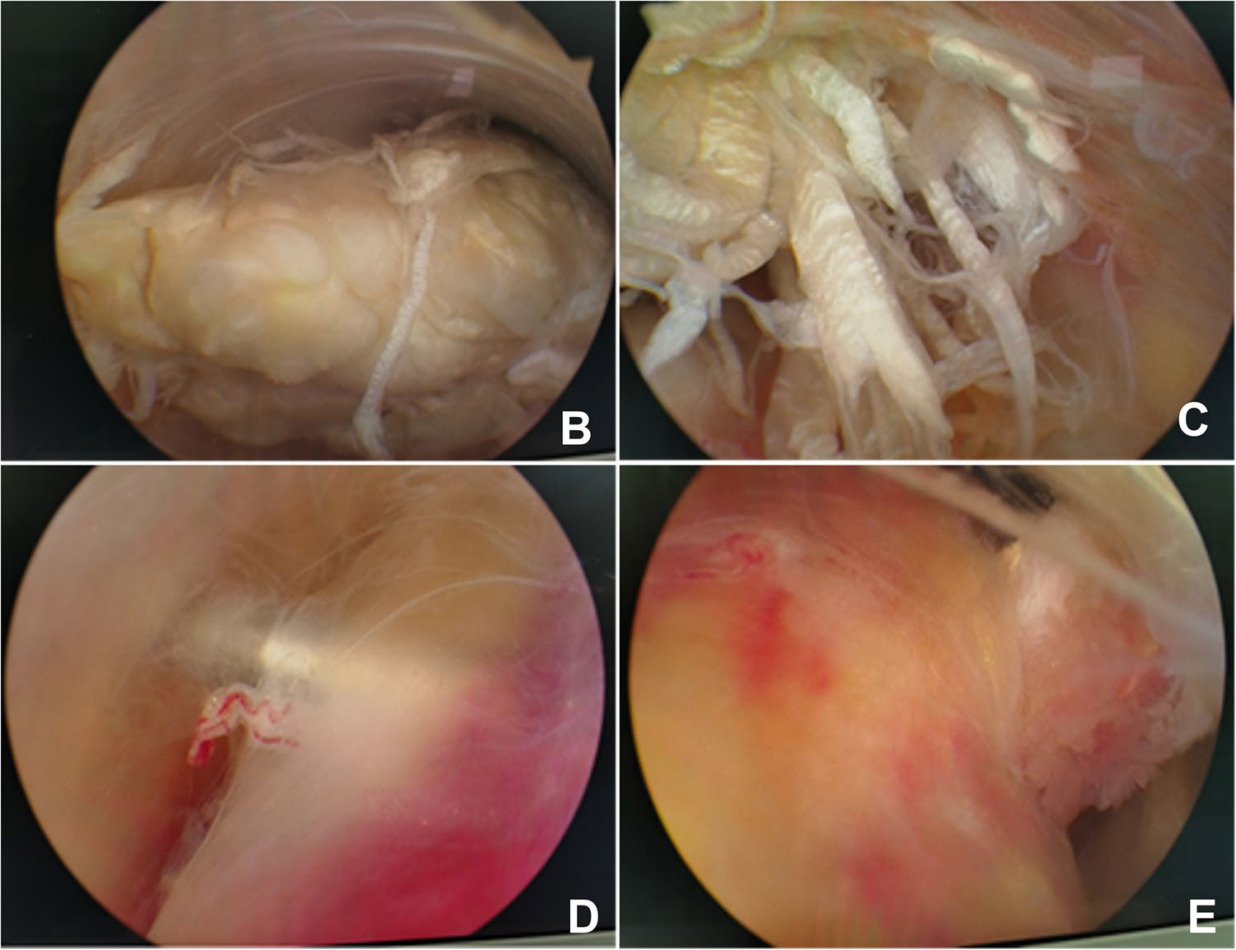
**B**-**C**: Exposure of the proximal and distal ends of the Achilles tendon under intraoperative endoscopic visualization; **D**: A proximal autologous tendon channel was established more than 2 cm away from the ruptured end of the Achilles tendon; **E**: A woven autologous tendon graft was implanted through the proximal channel

**Fig. 3:**
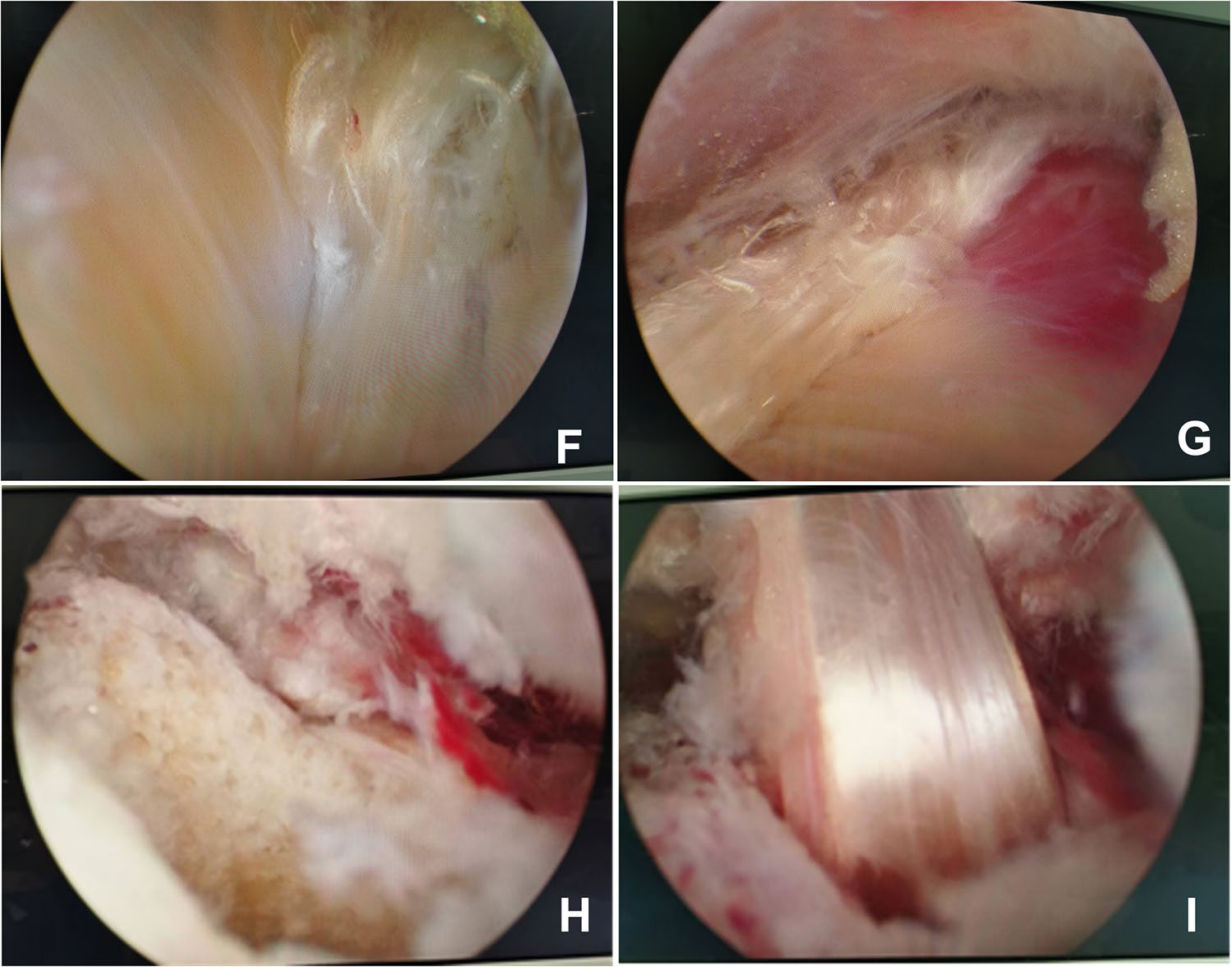
**F**-**G**: The autologous tendon was drawn distally from the medial and lateral aspects of the Achilles tendon; **H**-**I**: The calcaneal bone tunnel was established and the autologous tendon was introduced, with the interface screw being tightened

**Fig. 4:**
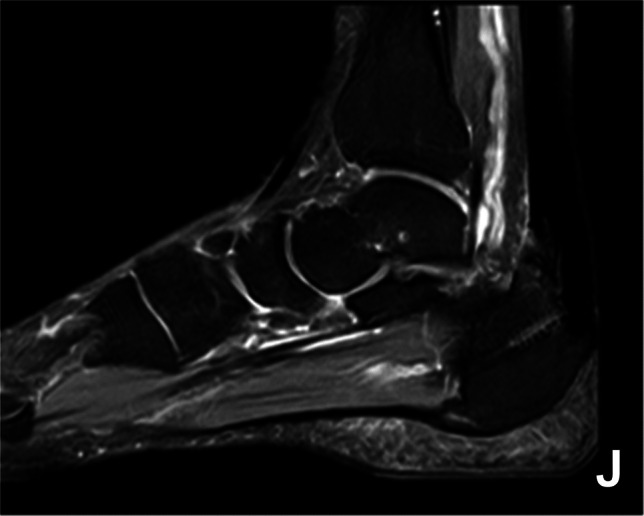
MRI images at 12 months postoperatively demonstrated that the continuity of the Achilles tendon has been reconstructed

**Fig. 5:**
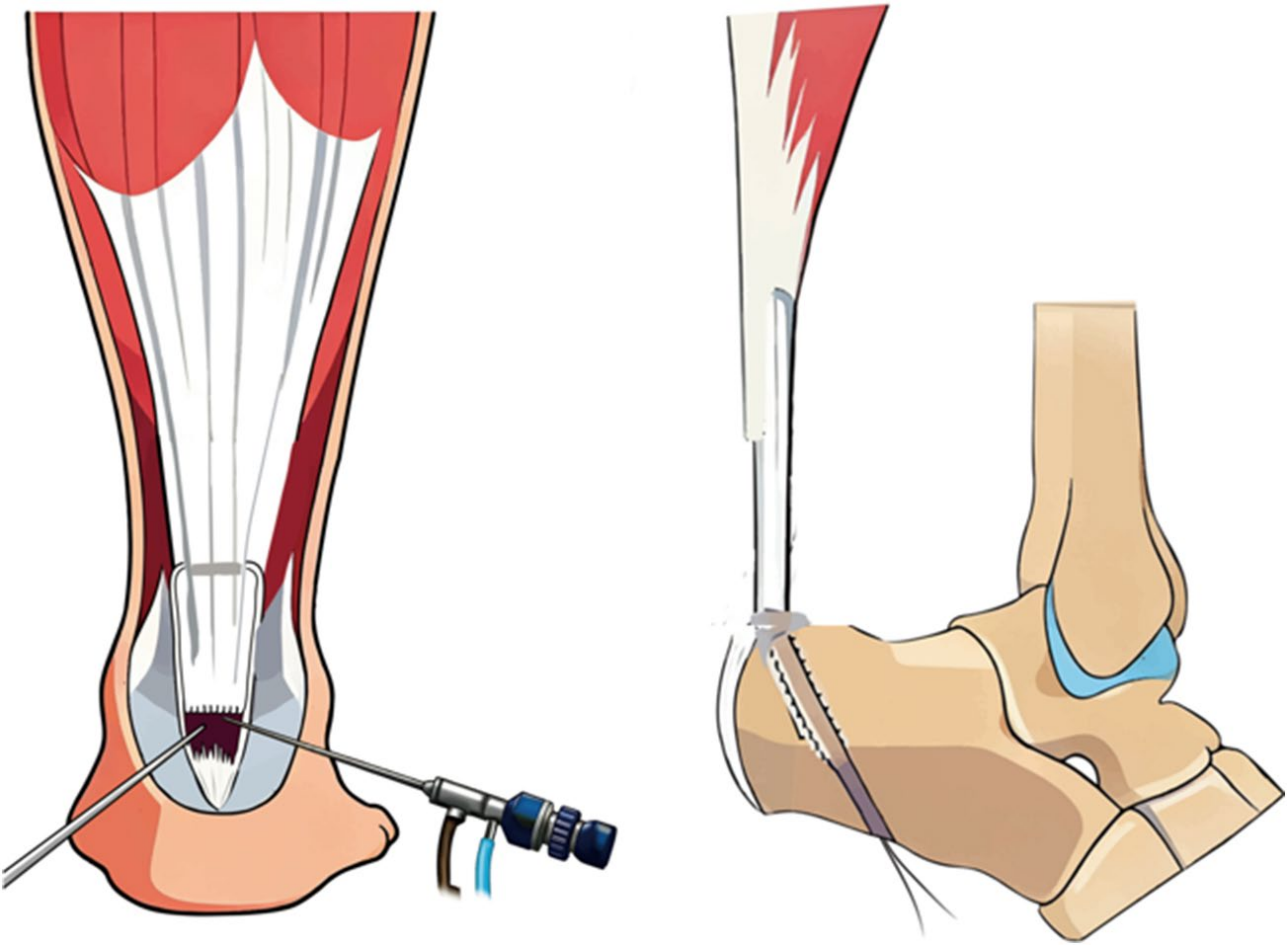
Postoperative anatomical pictures describing the graft positioning and channelling as well as the location of the fixation screw in the calcaneum

An endoscopic curettage was performed to clear haematoma from the Achilles tendon stump, remove degenerated tendon tissue at both proximal and distal ends, and hyperplastic synovium and calcifications, exposing the distal insertion of the Achilles tendon (Fig. 2B-C). If there were associated bony structural abnormalities such as Haglund's deformity or calcaneal spurs, excess bone should be resected with a burr. Subsequently, the ankle joint was positioned in slight plantar flexion (20°) to measure the length of the Achilles tendon defect was 6.6 ± 0.9 cm (range 5–8 cm). With the knee joint was flexed to 90˚, a 3 cm oblique incision was made 3 cm medial to the tibial tubercle, exposing the pes anserinus tendon and sharply incising the sartorius aponeurosis to identify and harvest the semitendinosus and gracilis tendons. The graft was woven and sutured at both ends with No.2 Vicryl tendon suture (Ethicon, Johnson and Johnson), the tendon length was measured to be 23.5 ± 2.2 cm (range 20–27 cm) and wrapped in moist saline gauze. The medial approach was used to insert the arthroscope and direct it distally. Under endoscopic visualization, a guide wire was inserted through the insertion point of the Achilles tendon towards the sole of the foot, followed by enlarging the hole with a 4.5 mm drill bit. Then, based on the diameter of the folded four-strand muscle tendon was 7(3) cm (range 6–12 cm), an appropriate drill bit was selected to establish the calcaneal bone tunnel. A puncture needle was inserted into the tendon tissue at a distance of ≥ 2 cm from the proximal stump under endoscopic visualization, establishing 0.5 cm incisional points on the medial and lateral sides of the Achilles tendon. Subsequently, a straight clamp was inserted through the transverse channel and appropriately expanded to establish a proximal tendon introduction channel (Fig. 2D). The graft was passed through the incision of the muscle tendon to produce equal medial and lateral lengths (Fig. 2E). The proximal transplanted muscle tendon is not sutured, and the autologous muscle tendon is suspended within the normal Achilles tendon tissue, while the distal graft was then threaded through the calcaneal tunnel and drawn out through the plantar wound. The ankle joint was held at 20° plantar flexion, with the graft was pulled through the calcaneal tunnel while maintaining maximum tension. The graft was secured with 6–8 mm interface screws, which was passed through the midline incision and into the calcaneus (Fig. 3H-I). The gastrocnemius squeeze test was examined the effect of Achilles tendon reconstruction by observing the plantar flexion movement of the ankle joint; a short leg plaster splint was used to fix the ankle in a 20° plantar flexion position (Figs. 4, 5).

### Postoperative management

Postoperative compression bandaging of the affected limb, along with symptomatic treatment measures such as ice application, swelling reduction, and pain relief. On the second postoperative day, the patients are encouraged to perform isometric and isotonic exercises for the ankle joint. The patients were transitioned from plaster to a tendon boot to begin progressive weight-bearing walking exercises at 6 weeks postoperatively. Crutches are usually continued to be used until the patient feels comfortable enough to move and exercise independently.

### Postoperative efficacy evaluation

All patients were assessed using a validated functional test battery at last postoperative follow-up, supplemented by additional clinical measures and patient-reported outcomes. Measure the maximum range of motion (ROM) of bilateral ankle joint and the maximum calf circumference. The clinical efficacy was assessed using the American Orthopaedic Foot & Ankle Society Ankle Hindfoot Scale (AOFAS-AH), Achilles tendon total rupture score (ATRS), visual analogue scale (VAS) for pain and Foot and Ankle Ability Measure (FAAM). The Arner-Lindholm criteria were used to estimate the rate of excellent outcomes. All functional scores and objective measurements were assessed by two independent blinded orthopaedic surgeons, and the mean values were used for analysis.

### Statistical analysis

Data analysis was conducted using SPSS 22.0 statistical software (IBM Corp., Armonk, NY, USA). The Shapiro–Wilk method is used to test for normal distribution of data, and the conforming quantitative data was expressed as mean ± standard deviation, while the comparison of observed indicators at different time points uses paired t-test. The non-normally distributed data were expressed as median (interquartile range, IQR), and non-parametric tests were used for analysis of outcomes. Wilcoxon's signed-rank test was used for comparison between the affected and the non-affected side. P < 0.05 was considered statistically significant. No sample size or statistical power calculations were performed as all available patients were included and no adequate control group could be identified.

## Results

All patients underwent endoscopic autologous muscle tendon suspension fixation without conversion to open surgery. The mean operative time was 62.91 ± 8.82 min (range: 45–80 min), and the average intraoperative blood volume was 15(11) ml (range: 5–35 ml). All 22 patients achieved primary wound healing, and there were no injuries to important structures such as blood vessels, nerves, and tendons during the operation. All patients received follow-up with a follow-up time of 16.23 ± 2.94 months (range: 12–23 months). During the follow-up, there were no complications such as re-rupture of the Achilles tendon or deep venous thrombosis (DVT) in the lower extremities.

At the last follow-up, the VAS score was significantly lower than before surgery, and both AOFAS-AH, ATRS, FAAM-ADL and FAAM-S improved compared to preoperative levels, with statistically significant differences (*P* < 0.05) (Table 2). The bilateral ankle range of motion examination, including the average angles of dorsiflexion and plantarflexion, and calf circumference was performed in patients at the last follow-up. There was no statistically significant difference between the healthy ankle and the affected one (*P* > 0.05) (Table 3). According to the Arner-Lindholm score: Excellent in 19 cases, good in 3 cases, with an excellent-to-good rate of 100% (22/22). All patients returned to their pre-injury level of activity 6 months post-operation, with 2 patients reporting weakness in single-leg heel raises, which partially improved after heel raise functional exercises.

**Table 2:** Comparison of VAS, AOFAS-AH, and ATRS scores at preoperative and last follow-up

Items	VAS	AOFAS-AH	ATRS	FAAM-ADL	FAAM-S
Preoperative	6(1)	60.64 ± 8.83	45.59 ± 5.85	44.73 ± 8.79	43.55 ± 7.14
Postoperative	1(0)	94.18 ± 3.91	93.18 ± 4.68	90.95 ± 4.62	88.27 ± 8.18
t/z	4.221	25.501	59.325	49.200	85.535
*P*	< 0.001	< 0.001	< 0.001	< 0.001	< 0.001

**Table 3:** Comparison of ankle joint range of motion and calf circumference between the affected and unaffected sides at the last follow-up

Items	Dorsiflexion Angle (°)	Plantarflexion angle (°)	Calf circumference(cm)
Affected side	13.23 ± 1.87	44.36 ± 1.65	35.61 ± 1.54
Healthy side	13.32 ± 1.94	44.50 ± 1.71	35.65 ± 1.55
Z	1.414	1.732	1.807
*P*	0.157	0.083	0.071

## Discussion

Achilles tendon rupture is a common condition in foot and ankle surgery, with an increasing incidence trend year by year [12, 13]. Following an acute Achilles tendon rupture, the ankle can still retain partial plantar flexion function due to the synergistic effect of other muscles, which is easily missed or misdiagnosed, ultimately developing into a chronic Achilles tendon rupture [5, 14, 15]. Currently, the option of surgical approach primarily determined by the size of the defect at the Achilles tendon after debridement. For patients with a smaller defect (< 3 cm), direct end-to-end suturing may be selected, while for those with a long defect, especially those > 6 cm, treatments such as V–Y advancement, muscle flap rotation, tendon transposition, and free tendon grafting are often required [4, 14, 16–19]. However, the selection of surgical approach remains controversial, and there is currently no unified standard. In our study, we present a small retrospective clinical series of patients who underwent semitendinosus and gracilis suspension fixation for reconstruction of chronic Achilles tendon ruptures and have overall favourable outcomes.

The flexor hallucis longus transfer is a representative method for treating chronic Achilles tendon rupture with long segment defects [20, 21]. While this procedure offers the advantage of mimicking the biomechanical properties of the Achilles tendon, it simultaneously weakens the flexor force of the big toe, compromises stability, and consequently impacts the patient's balance and gait. FHL transfer has been reported to result in a 60% reduction in hallux plantarflexion strength compared with the contralateral side [22]. Jiang et al. [7] applied autologous gracilis and semitendinosus tendon bridging to repair Myerson type III chronic Achilles tendon rupture, and all 7 patients achieved good clinical effects with no complications. However, this open surgical technique carries disadvantages including significant trauma, prolonged recovery, and potential wound complications. In a study on risk factors for wound complications following Achilles tendon surgery, 10.4% (17/164) of patients experienced wound complications; among those with risk factors such as diabetes, smoking, or steroid use, this proportion can reach up to 42.1% [23]. Endoscopic techniques have become a research hotspot for the treatment of chronic Achilles tendon rupture due to the advantages of minimally invasive treatment. Endoscopy-assisted techniques are an excellent option for reducing wound complications, particularly for elderly patients who are at increased risk of postoperative wound dehiscence and infection. The endoscopic technique provides good functional improvement with a high level of patient satisfaction in addition to the advantages of minimally invasive surgery. Vega et al. [10] performed flexor hallucis longus tendon transfer under arthroscopy for the treatment of chronic Achilles tendon rupture, including 22 patients with an average defect range of 6.3 cm, a follow-up time of 30.5 months, and an AOFAS score improved from 55 points preoperatively to 91 points postoperatively. Nilsson [24] reported 22 patients who underwent endoscopic-assisted Achilles tendon reconstruction using semitendinosus autograft, with the patients evaluated at 12 months postoperatively. The median (range) ATRS in patient reports was 76 (45–99) and ATRA on the injured side was 60° (49°−75°), compared to 49.5° (40–61°) on the non-injured side, *P* < 0.001. Two patients had minor postoperative wound complications, which healed uneventfully after seven days of oral antibiotic treatment. One patient experienced a sural nerve injury. In present study, the mean AOFAS score increased from 60.64 preoperatively to 94.18 at the last follow-up; the median VAS score improved from 6 to 1, and the postoperative ATRS was 93.18. To date, no major wound or neurovascular injury complications have been reported in the current case series.

In this study, we employed the technique of autologous tendon suspension fixation under all endoscopy to treat chronic insertional Achilles tendon rupture, which has the following advantages: (1) Compared with traditional open surgery, the entire Achilles tendon reconstruction process only requires five to seven incisional wounds of about 0.5 cm, achieving minimally invasive treatment and minimizing the incidence of wound complications. During the follow-up of this group of patients, there were no cases of poor wound healing were observed. (2) Patients with Achilles tendon rupture often have concomitant insertional Achilles tendinopathy. While treating the Achilles tendon rupture, addressing the pathogenic factors of the Achilles tendinopathy in a single stage ensures postoperative recovery effectiveness and reduces the incidence of recurrent tendon rupture. (3) The entire procedure was completed under endoscopic visualization, allowing for clear identification of important blood vessels and nerve structures around the Achilles tendon, thus avoiding iatrogenic injuries. No cases of major vascular or neural damage occurred in this cohort. (4) This technique does not require extensive detachment of the Achilles tendon, effectively preserving peritendinous blood supply and facilitating rapid postoperative recovery. (5) The autologous semitendinosus and gracilis muscle were utilized for bridging repair avoids damaging functionally critical autologous tendons. The semitendinosus and gracilis tendons are accessory stabilizers and flexors of the knee, and their removal is associated with minimal donor-site morbidity. No significant deficits in knee strength, stability, or range of motion have been reported in the literature. Patients in this study reported no subjective weakness or pain at the donor site, and all patients returned to their preinjury activity level within three months. Therefore, autologous harvest of semitendinosus and gracilis tendons is a safe and reliable option for Achilles tendon reconstruction with negligible functional impact. (6) The proximal end of the transplanted autologous tendon is secured with suspension fixation, while the distal end is stabilized with interface screws. This approach effectively ensuring the strength of the reconstructed Achilles tendon. Patients in this group can partially weight-bearing and walk under Achilles tendon boot protection six weeks postoperatively.

There is no standardized procedure for endoscopic Achilles tendon reconstruction surgery currently [13, 24, 25]. During surgical procedures, the distance between the proximal tendon graft channel and the Achilles tendon stump should be exceed 2 cm to ensure adequate suspension strength of the proximal graft tendon [1, 26], being too close may lead to failure of the suspension fixation. A distance that is too close may lead to failure of suspension fixation. Additionally, the tendon pathway should be estimated on the body surface first to ensure the tendon length in the calcaneal bone tunnel is approximately 2–3 cm. This is because the grafted tendon must be drawn out through the plantar surface, where abundant muscles exist. Excessively long tendons make it difficult to tighten when adjusting the tension, resulting in poor reconstruction of the Achilles tendon tension, and postoperative patients will experience weakness in heel raising. Two patients in this group reported weakness in single-leg heel raises, which is considered to be related to the excessive length of the distally transplanted autologous tendon. Although endoscopic techniques are attractive due to their advantages as minimally invasive surgery, surgeons should keep in mind the potential complications associated with tendon harvesting, tunnel drilling, or screw fixation, and should pay attention to technical details to avoid them. A fresh frozen cadaveric study indicates that in, graft fixation with two different interference screw insertion angles for IAT reconstruction exhibited equivalent biomechanical properties [27]. Intraosseous calcaneal tunnel collapse or rupture is another potential complication, which may be caused by improper tunnel drilling or improper screw fixation. Although direct endoscopic observation may be helpful, we recommend using a cannulated drill to guide the K-wire previously introduced in the desirable position. Additionally, appropriate screw size and direction must be considered and contemplated during introduction into the tunnel.

This study also has limitations: (1) the number of cases is small, lacking prospective randomized controlled trials; (2) the learning curve is long, and there are no natural cavities around the Achilles tendon, requiring the surgeon to master skilled endoscopic cavity creation techniques; (3) there is a lack of comparative studies with other surgical methods; (4) there was no comparison tension or strength with the contralateral limb. Future studies are required to assess the mechanical aspects of the described techniques.

## Conclusion

In summary, the favourable clinical results obtained in the short-term follow-up of this study indicate that endoscopic autologous tendon bridge repair is a viable surgical option for the treatment of chronic insertional Achilles tendon rupture.

## Data Availability

No datasets were generated or analysed during the current study.
